# Occupational Differences in Psychological Distress Between Chinese Dentists and Dental Nurses

**DOI:** 10.3389/fpsyg.2022.923626

**Published:** 2022-07-01

**Authors:** Yingying Zhang, Li Yan, Huiqing Long, Lu Yang, Jing Wang, Yiyun Liu, Juncai Pu, Li Liu, Xiaogang Zhong, Jin Xin

**Affiliations:** ^1^Key Laboratory of Psychoseomadsy, Stomatological Hospital of Chongqing Medical University, Chongqing, China; ^2^Chongqing Key Laboratory of Oral Diseases and Biomedical Sciences, Chongqing, China; ^3^School of Public Health and Management, Chongqing Medical University, Chongqing, China; ^4^NHC Key Laboratory of Diagnosis and Treatment on Brain Functional Diseases, The First Affiliated Hospital of Chongqing Medical University, Chongqing, China; ^5^Chongqing Stomatological Association, Chongqing, China

**Keywords:** psychological distress, General Health Questionnaire, dentists, nurses, occupational differences

## Abstract

**Background:**

Doctors and allied health professionals are facing serious mental health issues, which have received widespread attention. This study aimed to explore the occupational differences in psychological distress between Chinese dentists and dental nurses.

**Materials and Methods:**

The data was collected from a cross-sectional study conducted by the Chongqing Stomatological Association. Medical personnel involved in this survey were invited to complete a battery of self-administrated questionnaires, specifically the General Health Questionnaire-12, Maslach Burnout Inventory, and career choice regret scale. Data on demographic characteristics and working conditions were also collected. The results of these questionnaires were analyzed with SPSS (version 23.0). Univariate and multivariable analyzes were conducted to explore the influencing factors.

**Results:**

A total of 3,020 valid questionnaires, including 1,855 dentists and 1,165 dental nurses, were collected from 11 provinces of China. In general, 23.8% of responders exhibited psychological distress. The rate of dentists was 25.7%, and that of dental nurses was 20.8%. The prevalence was 4.9% higher in dentists than in dental nurses (*P* < 0.05). The multivariable analysis showed that factors associated with psychological distress for dentists were lower income, burnout, high job stress, career-choice regret, and lack of sufficient personal time, and that for dental nurses were age, lower income, longer working hours per week, burnout, high job stress, low job satisfaction, lack of sufficient personal time, and poor medical environment.

**Conclusion:**

The prevalence of psychological distress was relatively high among dental medical staff, and dentists showed a higher prevalence than dental nurses. Nurses have more risk factors for psychological distress than dentists. These results indicate that it is necessary to monitor the mental health status of dental medical staff and implement accurate strategies for dentists and dental nurses to promote their physical and mental health.

## Introduction

Mental health is a significant proposition related to social, psychological, behavioral, and biological factors (Zhou et al., 2018). It can be evaluated by the different psychiatric diagnoses, such as depression, anxiety, and schizophrenia (Hoeymans et al., 2004). With the rapid development of modern society, the above series of psychological distress not only can lead to insomnia and suicidal tendencies but also seriously affect physical health, such as hypertension (Grimsrud et al., 2009), diabetes (Sorsdahl et al., 2018), cardiac disease (Kapfhammer, 2011; Kinley et al., 2015), and even cancer (Mathé, 1996; Braczkowski et al., 1999; Kruk and Aboul-Enein, 2004).

The characteristics of high risk in the medical industry make medical personnel a very stressful profession. The long-term night shift, high workload, and long working hours predispose medical staff to experience negative emotions at work (Lawrence, 1989; Caplan, 1994), which may produce a detrimental effect on their work performance and daily life (Firth-Cozens and Greenhalgh, 1997; West et al., 2009). Numerous studies have shown that psychological distress is common among medical staff (Xu and Zhao, 2006; Wang et al., 2010; Zhou et al., 2017, 2018). For example, a survey of Chinese neurologists showed that 37.8% of respondents experienced psychological distress, which is independently associated with lower income, irregular working hours, high job stress, and low job satisfaction (Zhou et al., 2017). Another study showed that about two-thirds (69%) of American pediatric critical care physicians who experienced severe burnout screened positive for psychological distress (Shenoi et al., 2018), suggesting that job burnout may be a risk factor for psychological distress.

Medical personnel mainly comprise doctors and nurses. Although they work in the same environment, their mental health status may be different due to some distinctions in age, educational background, economic income, working years, and clinical duties (Panagopoulou et al., 2006; Senter et al., 2010; Thomas et al., 2014). Indeed, previous studies have indicated that the mental health of doctors and nurses significantly differs, but the results of these studies have varied. For example, studies about the prevalence of psychiatric distress in a general hospital in Italy or among dialysis healthcare workers have revealed that nurses have a higher prevalence of psychiatric distress than doctors (Klersy et al., 2007; Renzi et al., 2012), while others (e.g., the study of medical staff in a neonatal intensive care unit or in oncology) suggested that doctors are in worse mental health status than nurses (Chan et al., 2015; Grace and VanHeuvelen, 2019). These inconsistent results may be related to different influencing factors (e.g., work-life conflict, irregular work hours, work pressure, or social support) in different medical specialties (Grace and VanHeuvelen, 2019).

Dentistry has many stress elements in clinical practice, such as a noisy medical environment, difficult work postures, prolonged surgical procedures, narrow operating space, and a desire for technical perfection (Song et al., 2017). Dental medical staff members are likely to experience mental health issues after long-term exposure to these stress factors; further, dentists and dental nurses generally cooperate as a dyad, thus skilled and smooth collaboration is vital for efficient job performance in dental practice (Hakanen et al., 2014). Their relationship is closer compared with other specialties, which may predispose them to affect each other’s workload and exchange negative emotions (Westman et al., 2011; Hakanen et al., 2014), ultimately affecting their medical behavior. There are few studies that have focused on the comparison between dentists and dental nurses in psychological distress. Therefore, this study aimed to survey the occupational differences in psychological distress between Chinese dentists and dental nurses and explore respective influencing factors. It is hoped that our study can contribute to the development of accurate strategies to promote the physical and mental health of dental medical staff.

## Materials and Methods

### Study Design and Data Collection

The data was collected from the cross-sectional survey conducted by the Chongqing Stomatological Association between February and March 2021 in 11 provinces of China. We adopted convenience sampling to recruit the participants. Firstly, the Chongqing Stomatological Association contacted the directors of stomatology hospitals, dental departments in general hospitals and dental clinics via WeChat (a free messaging and calling app), telephone, or E-mail and invited them to participate in this survey. Secondly, if the director agreed to participate, dentists and dental nurses in their department were invited to participate in this study. Then, we sent self-administered questionnaires to them. The front page of the questionnaire introduced the background and purpose of the survey. The rights and potential risks to participants were also explained. Participation was anonymous and voluntary. If the participants returned the questionnaires, informed consent of participants was presumed. To ensure the accuracy of the data, we evaluated the quality of all questionnaires, and the exclusion criteria were as follows: (1) the same answer was chosen for the whole questionnaire item, and (2) only one questionnaire was included if two or more consecutive questionnaires had identical answers in the same working unit, and the other identical questionnaires were defined as invalid. Ethical approval was granted by the Ethics Committee of Stomatological Hospital of Chongqing Medical University (No. 2021-5).

### Survey Questionnaire

The self-administered questionnaires were constructed based on the questionnaire star platform^1^, a free platform widely used for surveys in China that creates an internet link to the questionnaire content. The staff of the Chongqing Stomatological Association sent the link to the directors who agreed to participate in this survey. Then, the director sent it to the dentists and dental nurses in their departments to invite them to participate. We implemented some settings for this questionnaire: (1) only the participants who completed all questions could submit the survey successfully to reduce the missing rate; otherwise, the incomplete questions were indicated, requiring participants to answer all questions, and (2) every internet protocol address could only be submitted once to ensure the uniqueness of each questionnaire.

The self-administered questionnaire comprised five parts. The first part was demographic characteristics, including gender, age, education, income, working years, hours worked per week, marital status, children status, commuting time, types of medical institution and major, and whether undertake teaching tasks.

The second part was the Chinese version of the General Health Questionnaire, composed of 12 items. It is widely used to assess mental health status (Zhong et al., 2021), and has also been successfully applied to Chinese civil servants, adolescents, and university students (Li et al., 2009; Ye, 2009; Liang et al., 2016). Each item is used to obtain a dichotomous score based on a conventional bimodal scoring method (0-0-1-1), rated on a 4-point scale. Participants are considered to have psychological distress if their total score is ≥4 (Haoka et al., 2010).

The third part was the Chinese version of the Maslach Burnout Inventory consisting of 22 items, which is used to measure the frequency and intensity of burnout in the allied health professions (Maslach et al., 2001). This Inventory measured three domains (emotional exhaustion, depersonalization, and personal accomplishment) of burnout using a 7-point Likert-type scale (range: 0–6). Having high levels of job burnout is considered if a responder scored ≥27 on emotional exhaustion or ≥10 on depersonalization, and a score of ≤33 is considered to indicate low personal accomplishment (Schaufeli et al., 2001; Rotenstein et al., 2018).

The fourth part adopted the Consultants’ Mental Health Questionnaire which was used to evaluate sources of job stress and satisfaction (Ramirez et al., 1996). A score of 0–4 indicates “not at all” and “extremely stressful/satisfying”, respectively. If a responder scored ≥3 on job stress and satisfaction questions, they were considered to have high levels of overall stress and satisfaction, respectively, whereas a score of ≤1 was considered low levels of overall stress and satisfaction, respectively (Teasdale et al., 2008).

The last part included seven questions as follows: (1) Would you choose to become a doctor/nurse again if given an opportunity? (2) Do you wish your child to be a doctor/nurse? (3) Do you have enough personal time? (4) What do you think of your current doctor-patient relationships? Each of the above questions was rated on a 3-point Likert scale as yes, neutral, or no. (5) Have you experienced hospital violence events? (6) Have you experienced a medical dispute? (7) Have you experienced an operation accident or misdiagnosis? The latter three questions were rated on a 3-point Likert scale (never, 1–3, or ≥4 times).

### Statistical Analysis

We performed all analyzes with SPSS version 23.0 (IBM Corp., Armonk, NY, United States). Qualitative data were summarized as frequencies and percentages, while quantitative data were summarized as means with standard deviation (SD) or medians with interquartile ranges depending on the distribution of the data. A univariate analysis was conducted by Chi-square tests or Fisher’s exact test. We adopted univariate logistic regression to screen variables and binary logistics (enter model, backward elimination model, and forward elimination model) to explore the potential influencing factors of psychological distress. The variables were considered to have no or slightly collinearity with the variance inflation factor (VIF) less than 10. *P* < 0.05 was considered statistically significant.

## Results

A total of 3,128 respondents from 180 stomatology hospitals, dental departments in general hospitals, and dental clinics in 11 Chinese provinces completed a battery of self-administered questionnaires by the end of March 2021. We excluded 99 invalid questionnaires with the same answers for the whole questionnaire items, and nine invalid questionnaires where two or more consecutive questionnaires had the same answers in the same working unit. Finally, 3,020 valid questionnaires (the effective rate was 96.55%, 3020/3128) were included in the subsequent analysis. A self-administered Chinese version of the questionnaire was adopted in this study, and the Cronbach’s α coefficient of the General Health Questionnaire-12 was 0.89. Cronbach’s α coefficient of the Maslach Burnout Inventory-22 was 0.74. The Cronbach’s α coefficient of the Hospital Consultants’ Job Stress and Satisfaction Questionnaire were 0.94 and 0.96, respectively.

### Participants Characteristics

Descriptive statistics of the demographic variables is shown in Table 1. Generally, about 61.7% of dentists and 99.1% of dental nurses were female. A total of 46.6% of dentists and 1.1% of dental nurses held doctorate or master’s degrees. For dentists, 31.8% worked >45 h/week, with 21.9% of them earning a monthly salary of <5,000 CNY. The percentage of dental nurses working >45 h/week was 22 and 37.3% of them earned a monthly salary of <5,000 CNY. Moreover, 60.9% of dentists and 72.5% of dental nurses worked in stomatology hospitals.

**TABLE 1:** Demographic characteristics.

Variables	Doctor	Nurse	*χ^2^*	*P*	Effect size
		
	N (%)	N (%)			
**Gender**			552.816	*P <* 0.001	0.428
Male	711 (38.3)	10 (0.9)			
Female	1144 (61.7)	1155 (99.1)			
**Academic degree**			759.495	*P* < 0.001	0.501
Doctor	137 (7.4)	0 (0)			
Master	727 (39.2)	13 (1.1)			
Undergraduate	828 (44.6)	831 (71.3)			
College and below	163 (8.8)	321 (27.6)			
**Age**			135.179	*P* < 0.001	−0.212
< 35 years old	1071 (57.7)	913 (78.4)			
≥ 35 years old	784 (42.3)	252 (21.6)			
Technical title			441.500	*P* < 0.001	0.382
Junior	815 (43.9)	948 (81.4)			
Intermediate	690 (37.2)	195 (16.7)			
Senior	350 (18.9)	22 (1.9)			
**Monthly income**			520.839	*P* < 0.001	0.415
< 5000 CNY	407 (21.9)	435 (37.3)			
5000–10000 CNY	683 (36.8)	689 (59.1)			
> 10000 CNY	765 (41.2)	41 (3.5)			
**Years of service**			92.146	*P* < 0.001	0.175
< 10 years	1172 (63.2)	837 (71.8)			
10–20 years	368 (19.8)	268 (23.0)			
> 20 years	315 (17.0)	60 (5.2)			
**Hours worked per week**			47.975	*P* < 0.001	0.126
< 45 h	1265 (68.2)	907 (77.9)			
45–55 h	421 (22.7)	218 (18.6)			
> 55 h	169 (9.1)	40 (3.4)			
**Marital status**			11.078	*P* = 0.011	0.061
Single	317 (17.1)	250 (21.5)			
Couple	226 (12.2)	133 (11.4)			
Married	1261 (68.0)	761 (65.3)			
Divorced or widowed	51 (2.7)	21 (1.8)			
**Whether have children**			1.159	*P* = 0.282	−0.020
No	753 (40.6)	496 (42.6)			
Yes	1102 (59.4)	669 (57.4)			
**Daily visits**			271.906	*P* < 0.001	0.300
< 10 patients	734 (39.6)	206 (17.7)			
10–20 patients	791 (42.6)	494 (42.4)			
20–30 patients	209 (11.3)	206 (17.7)			
> 30 patients	121 (6.5)	259 (22.2)			
**Tube bed**			4.940	*P* = 0.026	0.040
No	1662 (89.6)	1013 (87.0)			
Yes	193 (10.4)	152 (13.0)			
**Organization type**			44.514	*P* < 0.001	0.121
Stomatology hospitals	1130 (60.9)	845 (72.5)			
Dental departments in general hospitals	619 (33.4)	262 (22.5)			
Dental clinics	106 (5.7)	58 (5.0)			
**Professional type**			30.329	*P* < 0.001	0.100
General practice	774 (41.7)	557 (47.8)			
Oral medicine	428 (23.1)	234 (20.1)			
Maxillofacial surgery	184 (9.9)	155 (13.3)			
Prosthodontics	189 (10.2)	80 (6.9)			
Implant dentistry	75 (4.0)	39 (3.3)			
Orthodontics	205 (11.1)	100 (8.6)			
**Commute time**			58.064	*P* < 0.001	0.139
< 15 min	401 (21.6)	143 (12.3)			
15–30 min	766 (41.3)	469 (40.3)			
30–45 min	359 (19.4)	256 (21.9)			
45–60 min	208 (11.2)	189 (16.2)			
> 60 min	121 (6.5)	108 (9.3)			
**Undertake teaching tasks**			152.041	*P* < 0.001	0.224
Yes	776 (41.8)	234 (20.1)			
No	1079 (58.2)	931 (79.9)			
**Burnout**			10.194	*P* = 0.001	−0.058
No	1319 (71.1)	890 (76.4)			
Yes	536 (28.9	275 (23.6)			
**High job stress**			25.182	*P* < 0.001	−0.091
No	1481 (79.8)	1013 (87.0)			
Yes	374 (20.2)	152 (13.0)			
**Career choice regret**			3.340	*P* = 0.068	0.033
Other	1410 (76.0)	851 (73.0)			
Won’t be a doctor/nurse	445 (24.0)	314 (27.0)			
**Sufficient personal time**			64.639	*P* < 0.001	0.146
Yes	296 (16.0)	256 (22.0)			
Almost	827 (44.5)	612 (52.5)			
No	732 (39.5)	297 (25.5)			
**Medical environment**			213.801	*P* < 0.001	0.266
Good	252 (13.6)	384 (33.0)			
Normal	1181 (63.7)	681 (58.5)			
Bad	422 (22.7)	100 (8.5)			

*P (probability, value according to Chi-square test).*

### Prevalence Comparison of Psychological Distress

Table 2 showed that about 23.8% of the participants exhibited psychological distress, whereas the rate of dentists was 25.7%, and that of dental nurses was 20.8%. The prevalence of psychological distress in dentists was 4.9% higher than in nurses. The difference was statistically significant (*P* < 0.05).

**TABLE 2:** Prevalence of psychological distress.

Variables	Doctor	Nurse	χ^2^	*P*	Effect size
		
	N (%)	N (%)			
**Psychological distress**			9.097	*P* = 0.003	0.055
Yes	476 (25.7)	243 (20.8)			
No	1379 (74.3)	922 (79.2)			

*P (probability, value according to Chi-square test).*

### Univariate Analysis of Psychological Distress

The univariate analysis was performed to assess the relationships among personal and professional characteristics, burnout, job stress, job satisfaction, career-choice regret, etc., with psychological distress. The results revealed that the correlated factors of psychological distress for dentists were lower income, longer working hours per week, burnout, high job stress, lower job satisfaction, career-choice regret, don’t wish their child to be a doctor, lack of sufficient personal time, poor medical environment, experiencing violent events at the hospital, and medical disputes. Psychological distress for dental nurses was associated with older age, lower income, longer working hours per week, types of medical major, commuting time, undertaking teaching tasks, burnout, high job stress, lower job satisfaction, career-choice regret, don’t wish their child to be a doctor, lack of sufficient personal time, poor medical environment, experiencing violent events at the hospital, medical disputes, and operation accident (all *P* < 0.05; Supplementary Table 1). The results for univariate logistic regression are shown in Supplementary Table 2.

### Multivariable Analysis of Psychological Distress

The multivariable analysis revealed that lower income, burnout, high job stress, career-choice regret and lack of sufficient personal time were the risk factors for psychological distress in dentists. For dental nurses, the risk factors were age (older than 35 years), lower income, longer working hours per week, burnout, high job stress, low job satisfaction, lack of sufficient personal time, and poor medical environment. Among them, high job stress was the risk factor with the highest odds ratio (OR) of psychological distress for both dentists and dental nurses. Additionally, lower income, burnout, high job stress, and lack of sufficient personal time were the common risk factors for both dentists and dental nurses. With the increase in monthly income and personal time, the risk of psychological distress decreased (all *P* < 0.05; Table 3). The results for three types of logistic models and variance inflation factor (VIF) are shown in Supplementary Table 3.

**TABLE 3:** Multivariable analysis of psychological distress.

Variables	Psychological distress			
	
	Doctor		Nurse	
		
	OR (95% CI)	*P*	OR (95% CI)	*P*
**Age ≥ 35 years old**	–	–	2.069 (1.429–2.995)	<0.001
**Monthly income**		0.001		0.006
<5,000 CNY	1 (Reference)	–	1 (Reference)	–
5,000–10,000 CNY	0.694 (0.516–0.933)	0.016	0.654 (0.469–0.913)	0.013
>10,000 CNY	0.558 (0.415–0.75)	<0.001	0.279 (0.101–0.768)	0.013
**Hours worked per week**		–		0.015
<45 h	–	–	1 (Reference)	–
45–55 h	–	–	1.493 (1.025–2.175)	0.037
>55 h	–	–	2.433 (1.127–5.253)	0.024
**Burnout**	2.171 (1.699–2.773)	<0.001	1.772 (1.238–2.536)	0.002
**High job stress**	3.017 (2.306–3.946)	<0.001	3.37 (2.233–5.086)	<0.001
**Low job satisfaction**	–	–	1.742 (1.05–2.892)	0.032
**Career choice regret**	2.05 (1.594–2.637)	<0.001	–	–
**Sufficient personal time**		0.003		0.001
Yes	1 (Reference)	–	1 (Reference)	–
Almost	1.225 (0.843–1.782)	0.288	1.37 (0.843–2.228)	0.204
No	1.735 (1.188–2.533)	0.004	2.347 (1.385–3.976)	0.002
**Medical environment**		–		0.009
Good	–	–	1 (Reference)	–
Normal	–	–	1.741 (1.18–2.57)	0.005
Bad	–	–	2.2 (1.209–4.004)	0.01

*“–” indicates these variables were excluded in regression models, OR: odds ratio, P (probability, value according to logistic regression analysis).*

## Discussion

Over 3,000 dental medical staff members across almost all provinces in Western China were involved in this survey, representing the largest study analyzing psychological distress of dental medical staff ever reported. The results demonstrated that 23.8% of responders exhibited psychological distress, which is higher than that reported in a previous study among dentists and dental hygienists in Israel (11.5%) (Shacham et al., 2020).

The rate of psychological distress among dentists was 25.7%, which was lower than that in other medical specialties, such as neurologists in China (37.8%) (Zhou et al., 2017), trainee general practitioners in Spain (40.4%) (Ovejas-López et al., 2020), pediatric critical care physicians in the United States (30.5%) (Shenoi et al., 2018). Our study revealed that lower income, burnout, high job stress, career-choice regret, and lack of sufficient personal time were the risk factors for psychological distress in dentists. According to previous studies, frequent and prolonged night shifts, except for the above-mentioned factors, were also significant factors in other medical specialties, such as ICU physicians or psychiatrists (Zhou et al., 2017; Chen et al., 2021). These results indicated that more night shifts might greatly increase the incidence of mental illness in doctors. Dentists have little or no night shifts due to their professional characteristics, which may explain why dentists have a lower rate of psychological distress compared to other medical professions.

There are few studies that have focused on the prevalence of psychological distress among dentists. The studies have shown that dentists have a high prevalence of psychiatric symptoms, such as problematic depression (43.7%) and worry or anxiety (38%), which are mainly associated with the burden of acquiring new medical knowledge, conflicts with the patients, and the fear of medical accidents (Dunlap and Stewart, 1982; Song et al., 2017). It can be seen that the higher pursuit for medical technologies, poor doctor-patient relationships, and the occurrence of medical accidents can also affect the mental health of dentists.

Our research also showed that the rate of psychological distress in dental nurses was 20.8%, which was lower compared to previous studies. For example, the prevalence of psychological distress among nurses from a tertiary health institution in Nigeria was shown to be 44.1% (Okwaraji and Aguwa, 2014). Studies have also shown that marital status, level of education, the title of technician, and working years have been significantly associated with psychological distress in nurses (Okwaraji and Aguwa, 2014; Ghawadra et al., 2019), which is not consistent with our findings. For marital status, some studies have reported that single and widowed nurses have a higher level of stress, anxiety, and depression, perhaps because of social support and emotional balance provided by a spouse or partner (Perry et al., 2015; Badil et al., 2017; Ghawadra et al., 2019). Due to the progress of industrialization and modernization, China improved marriage equality, social security, and policy support, reducing the importance of the effect marriage has on health (Fu and Noguchi, 2016). As a result, marital status is not a major influencing factor in our study compared with Nigeria (Tatangelo et al., 2017; Rindner et al., 2020). Equal pay for equal work focuses on workload, which may explain why the level of education, the title of technician, and working years have little impact on the mental health of dental nurses (Silbersdorff and Schneider, 2019; Tibber et al., 2022).

Dentists have a 4.9% higher prevalence of psychological distress than nurses (*P* = 0.003). In addition to working hours and stress, income, job burnout and career choice regret have a great impact on the psychological distress in dentists. Dentists tended to be more educated, work longer hours per week, undertake more teaching tasks, and have higher levels of seniority and income compared to dental nurses. Study has shown that longer weekly hours and a higher medical degree (Doctor of Medicine or Doctor of Osteopathic Medicine) may increase the risk of burnout (Shanafelt et al., 2012), which may seriously affect doctors’ mental health. Additionally, dentists with higher education and job titles may have higher expectations for income, social status, and scientific research; hence, they may fail to keep their mental health when their expectations are not met (Lang-Runtz, 1984; Freeman et al., 1995; Rada and Johnson-Leong, 2004; Sancho and Ruiz, 2010). Compared to dentists, dental nurses are more susceptible to age and medical environment. Age of ≥35 years is a significant risk factor for psychological distress in dental nurses. Older nurses with abundant work experience may have a higher expectation for their personal value, while mismatched social status makes them feel that their efforts are not recognized, resulting in psychological imbalance (Almalki et al., 2012; Glerean et al., 2017). The slower acceptance for new knowledge and the insufficient opportunities for professional development may cause older nurses to feel anxious and disappointed and experience other undesirable emotions (Almalki et al., 2012; Asegid et al., 2014; Zhou et al., 2019). In clinical practice, doctors encourage a form of “teamwork” in which nurses are subordinates — nurses usually take the initiative to cooperate with doctors; this non-mutual collaborative work environment may have a greater impact on nurses (Campbell-Heider and Pollock, 1987; Chan and Huak, 2004). Moreover, some types of workplace stressors, such as the frequent rotation in nursing positions, patient care, and pressure from patients’ relatives, can also have an impact on nurses’ mental health (Lambert et al., 2004).

This study had several limitations. Firstly, there was selection bias in the study. Because we could not interview all dental staff, only 11 provinces were involved in this study, which may not be completely representative of the entire Chinese dental medical staff. Secondly, all data based on the analysis were derived from self-administered questionnaires. Some participants might not have been completely honest when reporting potential mental health issues; thus, the validity of the data collected might have been compromised. Thirdly, due to the cross-sectional nature of the survey, a causal relationship between the factors studied and the potential direction of influence for the associations we observed cannot be established. Fourthly, there may be many important factors related to psychological distress that were not shown in this survey. Therefore, further research on these factors in practice is needed. Eventually, the responsibilities between dentists and nurses who only work in hospitals or only work in dental clinics may be different, and more grouping analyzes are needed to solve this issue.

## Conclusion

The prevalence of psychological distress was relatively high among dental medical staff, and dentists showed a higher respective prevalence than dental nurses. Nurses have more risk factors for psychological distress than dentists. All these results indicate that it is necessary to monitor the mental health status of dental medical staff and implement accurate strategies for dentists and dental nurses to promote their physical and mental health.

## Data Availability Statement

The original contributions presented in this study are included in the article/Supplementary Material, further inquiries can be directed to the corresponding authors.

## Ethics Statement

The questionnaire and study protocol were reviewed and approved by the Ethics Committee of Stomatological Hospital of Chongqing Medical University (No. 2021-5). The front page of the questionnaire introduced the background and purpose of the survey, the rights and potential risks were informed to participants as well. Participation was anonymous and voluntary. If the participants returned the questionnaires, informed consent of participants was presumed.

## Author Contributions

YZ and XJ proposed the concept and design. LiY, HL, LuY, and JW collected the data. YZ, XZ, and XJ analyzed and interpreted the data and wrote the manuscript. YL, JP, and LL edited the manuscript. XJ and XZ provided funding and supervised the study. All authors reviewed and approved the manuscript prior to its submission.

## Conflict of Interest

The authors declare that the research was conducted in the absence of any commercial or financial relationships that could be construed as a potential conflict of interest.

## Publisher’s Note

All claims expressed in this article are solely those of the authors and do not necessarily represent those of their affiliated organizations, or those of the publisher, the editors and the reviewers. Any product that may be evaluated in this article, or claim that may be made by its manufacturer, is not guaranteed or endorsed by the publisher.

## Footnotes

1
https://www.wjx.cn/
